# A Cohort Study on the Outcome of Diabetic Foot Ulcers

**DOI:** 10.7759/cureus.48030

**Published:** 2023-10-31

**Authors:** Sriram Sanjeeviraj, Aswinkumar Subburaj, Smriti Aluri, Brahmma Rishy Thakku Sekar, Manik Jalan, Aleena Gilton Joseph

**Affiliations:** 1 General Surgery, Madras Medical College, Chennai, IND; 2 Surgery, Vinayaka Mission’s Research Foundation, Karaikkal, IND; 3 Surgery, Kakatiya Medical College, Warangal, IND; 4 Surgery, Government Sivagangai Medical College and Hospital, Sivagangai, IND; 5 Emergency Medicine, Tagore Hospital and Heart Care Centre, Jalandhar, IND; 6 Surgery, SK Hospital, Thiruvananthapuram, IND

**Keywords:** amputations., glycemic control, risk factors, healing rate, diabetic foot ulcer

## Abstract

Background: Diabetic foot ulcers (DFUs) represent a significant and challenging complication of diabetes mellitus, often leading to serious morbidity and a substantial burden on healthcare systems. The study was conducted with the objectives of evaluating the outcomes of DFUs.

Materials and methods: A cohort study was conducted to evaluate the outcomes of DFUs from May 2019 to May 2020 at a tertiary care hospital located in Chennai. The study included patients aged 18 to 90 years who were diagnosed with DFUs. Individuals with diabetic foot lesions (skin lesions such as fissures, abscess, cellulites) other than ulcers or those without diabetes were excluded. The data was collected from a total of 100 diabetic patients using systematic random sampling technique.

Results: The mean (SD) age of the study participants was 54.68 (6.72) years with males constituting 56% of the study population. Among 100 participants, 65% experienced healing while 35% did not. Logistic regression analysis showed that glycated haemoglobin (HbA1c) levels, age, and diabetes duration had significant effect on patient outcome. Logistic regression analysis showed that HbA1c levels, age, and diabetes duration had significant effect on patient outcome. Out of 12 patients with major amputation, seven (58.3%) survived, while out of 19 patients with minor amputations, 18 (94.7%) showed remarkably higher survival rate. Meanwhile, 100% survival rate was observed in patients with no amputation.

Conclusion: The study's comprehensive assessment of risk factors and their associations with healing outcomes provides essential knowledge for clinical practice. The study findings collectively support the optimization of interventions and strategies to prevent and manage DFUs, ultimately improving patient care and enhancing their quality of life. The study highlights the significance of glycemic control and limb preservation in DFU management.

## Introduction

Diabetic foot ulcers (DFUs) represent a significant and challenging complication of diabetes mellitus, often leading to serious morbidity and a substantial burden on healthcare systems [1-3]. The impact of DFUs extends beyond physical discomfort, affecting patients' quality of life, mobility, and overall well-being [4]. Despite advancements in medical science, the management of DFUs remains a complex and multidisciplinary endeavor [5]. Understanding the outcomes associated with these ulcers is pivotal for optimizing patient care and enhancing treatment strategies [6,7].

The prevalence of diabetes mellitus is rising globally, and India ranks among the countries with the highest diabetes burden. DFUs emerge as one of the most common and debilitating complications of diabetes, accounting for a considerable proportion of diabetes-related hospitalizations and healthcare expenditures [8,9]. The multifaceted nature of DFUs, encompassing aspects of neuropathy, ischemia, and infection, necessitates tailored therapeutic approaches [10,11].

DFUs have garnered attention due to their substantial contribution to increased morbidity, reduced quality of life, and escalating healthcare expenditures [12]. The deleterious consequences that accompany DFUs encompass not only localized infections and delayed wound healing but also more severe outcomes such as gangrene and, in certain instances, amputations [13]. The gravity of these outcomes underscores the need for meticulous investigation into the factors that influence the healing process, the potential for recurrence, the necessity for amputations, and the overall survival of individuals afflicted by DFUs [14].

This study addresses the urgent need to understand and tackle the issues surrounding DFUs in the Indian healthcare landscape. Despite the acknowledged prevalence and clinical significance of DFUs, there's a noticeable lack of comprehensive studies providing insights into outcomes, particularly in the Indian context. Understanding the factors contributing to healing or non-healing, the impact of interventions and risk factors is vital for effective management and prevention [15-17]. Given the increasing prevalence of diabetes, the study's insights into the outcomes of DFUs among patients in tertiary care hospital are crucial, with the potential to shape clinical practices, interventions, and policies to alleviate the DFU burden and improve the quality of life diabetic patients [18].

## Materials and methods

Study setting

This cohort study was conducted at the Department of General Surgery, Madras Medical College, Chennai, to evaluate the outcomes of DFUs from May 2019 to May 2020. The study included patients aged 18 to 90 years with DFUs and having glycated haemoglobin (HbA1c) level >6.5. 

Individuals with diabetic foot lesions other than ulcers or those without diabetes were excluded. The terms lesions here refers to the blisters or corns that occurs during diabetes. A total of 100 adult patients with DFUs were enrolled for this study. The study was conducted after obtaining approval from the institutional ethical committee (approval number: MC/MMC/IEC/270/2019). Informed consent was acquired from all study participants.

Sample size and sampling technique

The sample size was determined using the formula 4pq/d². The prevalence of healed diabetic ulcers from a prior study was found to be 60% [19]. Utilizing this information in the formula and allowing for a 10% margin of error, the calculated sample size was 100 with the power of the study of 80%. A systematic random sampling approach was employed. Every fifth patient with DFUs who reported to the department of general surgery was included in the study. Data collection continued until a minimum sample size of 100 participants was achieved.

Methodology

Diagnosis of peripheral neuropathy was based on clinical signs, symptoms, and objective measures such as insensitivity to a 10 g Semmes-Weinstein monofilament, absence of vibration perception using a 128 Hz tuning fork, and the lack of ankle reflexes. Ischemia diagnosis relied on bedside examination, looking for specific indicators like dry, shiny, hairless skin, brittle nails, and cool-to-touch skin, along with measurement of the ankle-brachial index.

For the purpose of this study, healing was defined as continuous viable epithelial covering over the previously open wound; persisting unhealed was defined as incomplete re-epithelialization of the wound; minor amputation was defined as amputation restricted to the foot, not impairing walking ability (transmetatarsal, tarsometatarsal, or Lisfranc’s amputation); major amputation was defined as amputation performed above the ankle level; and recurrence was defined as re-ulceration, typically occurring on the same foot.

Statistical analysis

Demographic data and diabetes duration were recorded. The associations between variables were tested using the χ² test and Fisher's exact test. Logistic regression analysis was applied for multivariate analysis to account for other variables when assessing the effect of each risk factor on DFU outcomes. Survival analysis was performed using the Kaplan-Meier survival test. In all statistical tests, a significance level of less than 0.05 was considered statistically significant.

## Results

The data was collected from 100 study participants with 100% response rate. There was no dropout in this study. The mean (SD) age of the study participants was 54.68 (6.72) years. Table 1 provides a comprehensive overview of the characteristics of these participants and the distribution of key variables.

**Table 1:** Characteristics of the study participants HbA1c: Glycated haemoglobin

Characteristics		Number	Percentage
Gender	Male	56	56
	Female	44	44
BMI	Normal	42	42
	Overweight	31	31
	Obese	27	27
Type of diabetes mellitus	Type 1	6	6
	Type 2	94	94
Treatment	Oral antidiabetics only	36	36
	Insulin only	35	35
	Both	28	28
	No treatment	1	1
HbA1c (%)	6.5-7	16	16
	7.1-8	31	31
	8.1-9	35	35
	>9	18	18
Infections	Present	48	48
	Absent	52	52
Smoking status	Smoker	18	18
	Non-smoker	82	82

The gender distribution among the participants was fairly balanced, with 56% identifying as male and 44% as female. The participants' BMI was also examined, revealing that 42% had a normal BMI (normal range is between 18.5 to 24.9), 31% were overweight, and an additional 27% fell into the obese category.

Regarding the type of diabetes mellitus, most participants (94%) were identified as having type 2 diabetes, while only 6% had type 1 diabetes. Treatment approaches varied among the participants, with 36% receiving oral antidiabetics, 35% undergoing insulin treatment exclusively, and 28% utilizing a combination of both methods. A small fraction (1%) did not receive any form of diabetes-specific treatment.

The study also assessed the participants' HbA1c levels, a crucial marker of long-term blood sugar control. Out of 100 patients, 16% had HbA1c levels in the range 6.5-7%, 31% of the patients had level 7.1-8%, 35% had level 8.1-9% and 18% had levels >9, respectively.

The presence of infections (assessed by the presence of pus and confirmed by culture followed by antibiotic sensitivity testing) in relation to DFUs was another aspect under investigation. Infections here refers to local infections and the treatment was provided based on antibiotic sensitivity profile of the individuals and hospital protocol. The study found that 48% of participants had infections (Wagner Grades 2 and 3) present, while the remaining 52% (Wagner Grade 1) were devoid of such infections

Smoking status was also examined, revealing interesting trends among participants. Among the participants, 18% were smokers and 82% were non-smokers.

Table 2 shows association between various risk factors and the healing status of the DFUs. The impact of age on the healing of DFUs was a significant finding of the study. Participants were grouped into three age categories: 25-45 years, 46-64 years, and ≥65 years. The results revealed that within the youngest age group (25-45 years), majority of cases (97.1%) resulted in healed ulcers, and only 2.9% had unhealed ulcers. In the middle age group (46-64 years), a higher percentage of unhealed ulcers (38%) was observed, while in the oldest age group (≥65 years), 93.7% of ulcers remained unhealed. The observed p-value of 0.001 indicates a significant association between age and ulcer healing status.

**Table 2:** Association between DFUs and its risk factors DFU: Diabetic foot ulcer; HbA1c: Glycated haemoglobin *P values < 0.05 and statistically significant

Risk factors	Groups	DFUs		P value
		Healed N (%)	Unhealed N (%)	
Age	25–45 years	33 (2.9)	1 (97.1)	0.001*
	46–64 years	31 (62)	19 (38)	
	≥65 years	1 (6.3)	15 (93.7)	
Diabetes mellitus duration	<5 years	26 (92.9)	2 (7.1)	0.001*
	5–10 years	31 (91.2)	3 (8.8)	
	>10 years	8 (21.1)	30 (78.9)	
HbA1c	6.5-7	16 (100%)	0 (0%)	0.001*
	7.1-8	22 (71%)	9 (29%)	
	8.1-9	24 (68.6%)	11 (31.4%)	
	>9	3 (16.7%)	15 (83.3%)	
Peripheral neuropathy	Present	12 (54.5)	10 (45.5)	0.244
	Absent	53 (67.9)	25 (32.1)	
Ulcer size	<1 cm	15 (100)	0 (0)	0.001*
	1–5 cm	48 (94.1)	3 (5.9)	
	>5 cm	2 (5.9)	32 (94.1)	
Smoking status	Smoker	7 (38.9%)	11 (61.1%)	0.010*
	Non-smoker	58 (70.7%)	24 (29.3%)	

The duration of diabetes mellitus also emerged as a noteworthy determinant of ulcer outcomes. Participants were categorized into three groups based on the duration of diabetes: <5 years, 5-10 years, and >10 years. Among those with diabetes for less than 5 years, the majority (92.9%) experienced ulcer healing, in contrast to a mere 7.1% with unhealed ulcers. Similar trends were observed in the 5-10 years duration group, where 91.2% exhibited healed ulcers. However, for those enduring diabetes for over 10 years, a substantial majority (78.9%) faced unhealed ulcers, while only 21.1% witnessed healing. The calculated p-value of 0.001 underscores the statistical significance of diabetes duration in relation to ulcer healing outcomes.

HbA1c levels were another vital parameter assessed in the study. In this study, all 16 patients with HbA1c level 6.5-7% showed 100% ulcer healing; in 31 patients with HbA1c level 7.1-8%, healing was observed in 22 (71%) and nine (29%) remained unhealed; in 35 patients with HbA1c level in the range 8.1-9%, 24 (68.6%) displayed healing and 11 (31.4%) were unhealed; and among the nine patients with HbA1c level >9, all the patients remained unhealed. The statistically significant p-value of 0.001 highlights the impact of HbA1c levels on ulcer healing.

Peripheral neuropathy, a common complication of diabetes, was also analyzed as a risk factor. Participants were classified based on the presence or absence of peripheral neuropathy. While a higher percentage of those with peripheral neuropathy experienced unhealed ulcers (45.5%), a significant proportion without peripheral neuropathy (32.1%) also had unhealed ulcers. The p-value of 0.244 suggests that the association between peripheral neuropathy and ulcer healing may not be statistically significant.

The size of the ulcers, another determinant, demonstrated intriguing outcomes. Ulcer size was categorized as <1 cm, 1-5 cm, and >5 cm. All participants with ulcers smaller than 1 cm experienced healing, while in the 1-5 cm category, the majority (94.1%) achieved healing, and only 5.9% healed in those ulcers with size >5 cm. Remarkably, among ulcers larger than 5 cm, 94.1% remained unhealed. The p-value of 0.001 denotes the substantial influence of ulcer size on healing outcomes.

Regarding smoking status, the incidence of unhealed foot ulcer was higher in diabetic patients who were smoking as compared to non-smokers (61.1% vs 29.3%;p=0.010). 

Table 3 presents the results of a logistic regression analysis. Age, an essential determinant, demonstrated an odds ratio of 0.99, suggesting a slight negative association with ulcer healing. The 95% confidence interval (CI) ranged from 0.95 to 1.01, implying that the odds of ulcer healing were not significantly impacted by age. The corresponding p-value of 0.145 underscores the lack of statistical significance in the association between age and ulcer healing.

**Table 3:** Logistic regression analysis for risk factors associated with healing of DFUs HbA1c: Glycated haemoglobin; DFU: Diabetic foot ulcer; CI: Confidence interval

Risk factors	Odds ratio	95% CI	P value
Age	0.99	0.95–1.01	0.145
Diabetes mellitus duration	0.92	0.81–1.14	0.201
HbA1c	1.35	1.04–1.78	0.045*
Peripheral neuropathy	1.08	0.88–1.38	0.347
Smoking	1.12	0.92-1.10	0.125
Ulcer size	1.05	0.79–1.34	0.165

Examining the duration of diabetes mellitus, the odds ratio was calculated at 0.92, indicating a modestly negative relationship with ulcer healing. The 95% CI spanned from 0.81 to 1.14, revealing that the odds of ulcer healing were not substantially influenced by the duration of diabetes. The p-value of 0.201 further supports the absence of statistically significant association between diabetes duration and ulcer healing outcomes.

HbA1c levels emerged as a particularly influential factor. The odds ratio stood at 1.35, suggesting a positive relationship between elevated HbA1c levels and the likelihood of unhealed ulcers. The 95% CI ranged from 1.04 to 1.78, indicating a certain degree of uncertainty in the estimation. The p-value of 0.045 signifies the statistical significance of HbA1c levels in relation to ulcer healing.

Peripheral neuropathy, a common diabetic complication, displayed an odds ratio of 1.08, implying a marginal positive association with unhealed ulcers. The 95% CI ranged from 0.88 to 1.38, signifying that peripheral neuropathy's impact on ulcer healing odds was not definitive. With a p-value of 0.347, the lack of statistical significance in this relationship is evident.

Ulcer size, another risk factor, exhibited an odds ratio of 1.05, suggesting a negligible positive influence on the likelihood of unhealed ulcers. The 95% CI spanned from 0.79 to 1.34, illustrating a certain degree of variability in the odds estimation. The p-value of 0.165 reinforces the absence of statistical significance in the association between ulcer size and healing outcomes.

Smoking, as a risk factor, displayed an odds ratio of 1.12, implying a marginal positive association with unhealed ulcers. The 95% CI ranged from 0.92 to 1.10 and shows that smoking impact on ulcer healing odds was not definitive. With a p-value of 0.125, the lack of statistical significance in this relationship is evident.

Table 4 provides a comprehensive insight into the outcomes of the study participants with DFUs. Of the 100 participants included in the study, 65% experienced a positive outcome, with their ulcers healing successfully. This represents a significant proportion of individuals who benefited from the interventions and care strategies employed during the study.

**Table 4:** Outcome of DFUs DFU: Diabetic foot ulcers

Outcome	Number	Percentage
Healed	65	65
Unhealed	35	35
Minor amputations	19	19
Major amputations	12	12
Recurrence	15	15
Death	6	6

However, it's important to note that 35% of the participants did not achieve healing, reflecting the challenges and complexities often associated with DFUs. A noteworthy aspect of the study was the occurrence of amputations, both minor and major. Among the participants, 19% required minor amputations as a measure to manage the ulcer's progression. Major amputations were required for 12% of the participants. Recurrence of DFUs was observed in 15% of participants. This finding underscores the challenges associated with long-term management and the need for ongoing monitoring and preventive measures even after healing has been achieved. Regrettably, 6% of participants in the study succumbed to the complications associated with DFUs.

The study focused on assessing the outcomes of DFUs, with a particular emphasis on the impact of different types of amputations (major and minor amputations). Among 12 patients who underwent major amputations, a significant proportion 7 individuals (58.3%) survived the procedure. Meanwhile, among the 19 patients who had minor amputations, 18 patients (94.7%) survived. However, all the patients who did not undergo any form of amputation were survived. This outcome underscores the significance of preserving limbs whenever possible and the positive impact it has on patient survival rates.

**Figure 1:**
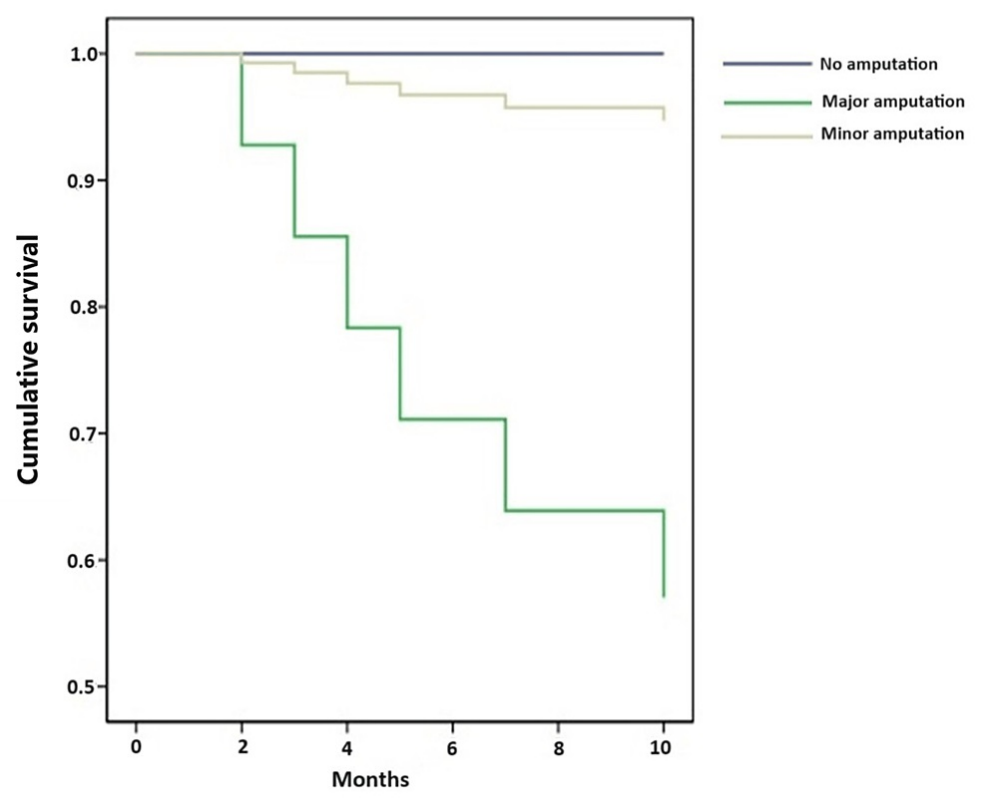
Survival rates among the study participants

The observed differences in survival rates were subjected to statistical analysis, revealing a statistically significant relationship (P = 0.034). Figure 1 illustrates the statistically significant differences in survival rates across the various groups.

## Discussion

The findings of this study provide crucial insights into the outcomes and determinants of DFUs within the Indian context. DFUs present substantial challenges to patients and healthcare systems, requiring comprehensive understanding for effective management and prevention strategies. The study's comprehensive investigation sheds light on the multifaceted nature of DFUs, from risk factors influencing healing outcomes to the impact of different amputation approaches on patient survival. The study's emphasis on DFUs is timely, considering the escalating prevalence of diabetes in India and its associated burden. DFUs, prevalent among diabetic individuals, contribute significantly to hospitalizations and healthcare expenditures [8,9]. The outcomes of DFUs extend beyond the physical realm, impacting patients' overall quality of life and mobility [4]. Therefore, deciphering the factors that influence their outcomes is imperative for developing interventions that mitigate the burden of DFUs. The study's outcomes reveal significant associations between various risk factors and ulcer healing status. Age emerged as a pivotal determinant, with older age groups exhibiting higher proportions of unhealed ulcers. This finding is consistent with previous studies that have highlighted the challenges faced by older individuals in achieving ulcer healing [20]. Diabetes duration played a crucial role in outcomes, with prolonged diabetes correlating with a higher likelihood of unhealed ulcers. This underscores the need for early interventions and stringent diabetes management to prevent the development of DFUs [21].

HbA1c levels, a key marker of glycemic control, exhibited a profound influence on DFU healing outcomes. Participants with lower HbA1c levels demonstrated a significantly higher proportion of ulcer healing, reaffirming the importance of tight glycemic control in preventing and managing DFUs [22]. Peripheral neuropathy, although not statistically significant in this study, remains a crucial factor influencing DFU outcomes [23]. Additionally, ulcer size emerged as a vital predictor of healing, with larger ulcers being associated with higher rates of unhealed outcomes. This highlights the need for early detection and intervention to prevent ulcer progression and larger wound sizes [24]. The logistic regression analysis further affirmed the impact of these risk factors on DFU outcomes. While age and diabetes duration did not demonstrate statistically significant associations with healing outcomes, elevated HbA1c levels were significantly correlated with unhealed ulcers. This finding underscores the role of glycemic control in shaping DFU outcomes [25]. Peripheral neuropathy, although marginally associated, aligns with existing literature that highlights its role in impeding ulcer healing [26]. Ulcer size, as revealed by the analysis, is a critical predictor of healing outcomes, reinforcing the importance of timely intervention to prevent ulcer progression [27,28].

The study's exploration of DFU outcomes also considered the impact of different types of amputations on patient survival. The findings indicate that among patients who underwent minor amputations, a notably higher percentage survived the procedure compared to those undergoing major amputations. This observation highlights the importance of preserving limbs whenever possible, aligning with the principles of limb salvage in managing DFUs [29,30]. Amputation was not offered, and it was based on clinical decision taken by the treating doctor. The observed statistically significant differences in survival rates across the various groups further validate the importance of the study's investigation. The study's findings hold the potential to inform clinical practice by emphasizing the impact of different amputation approaches on patient survival. These insights have the potential to guide treatment decisions, contributing to improved patient care and outcomes.

The study's single-center design and relatively small sample size could limit the generalizability of its findings. Additionally, other factors that might influence DFU outcomes, such as socio-economic status and patient adherence to treatment, were not fully explored in this study.

## Conclusions

This study contributes valuable insights into the outcomes of DFUs. The study's comprehensive assessment of risk factors and their associations with healing outcomes provides essential knowledge for clinical practice. These findings collectively support the optimization of interventions and strategies to prevent and manage DFUs, ultimately improving patient care and enhancing their quality of life.
